# Dynamic movement patterns of commensal rodents *Mastomys natalensis* and *Rattus rattus* : determining differential habitat use using Rhodamine B

**DOI:** 10.1002/ps.8435

**Published:** 2024-09-27

**Authors:** Herieth Mkomwa, Rhodes Makundi, Steven Belmain, Alfan A. Rija, Apia Massawe, Aurore Ponchon, Mwajabu Selemani, Marcela P.A. Espinaze, Sandra Telfer

**Affiliations:** ^1^ Department of Wildlife Management Sokoine University of Agriculture Morogoro Tanzania; ^2^ African Centre of Excellence for Innovative Rodent Pest Management and Biosensor Technology Development, Sokoine University of Agriculture Morogoro Tanzania; ^3^ Institute of Pest Management, Sokoine University of Agriculture Morogoro Tanzania; ^4^ Natural Resources Institute, University of Greenwich Kent UK; ^5^ School of Biological Sciences, Zoology Building, University of Aberdeen Aberdeen UK; ^6^ CEFE, Univ Montpellier, CNRS, EPHE, IRD Montpellier France

**Keywords:** *Mastomys natalensis*, *Rattus rattus*, Pest rodents, habitat preference, consumption rate and Tanzania

## Abstract

**BACKGROUND:**

Understanding movement patterns of rodent pests is essential for planning management strategies. Currently, for many rural village contexts, there is limited information on how rodents move between domestic and peridomestic areas, and the surrounding habitats. We investigated movement of the multimammate rat, *Mastomys natalensis* and the black rat, *Rattus rattus* in nine villages in Kilombero District, Tanzania. We used Rhodamine B (RhB) baits placed inside houses (*R. rattus* preferred habitat) in five villages and placed outside (*M. natalensis* preferred habitat) in four villages.

**RESULTS:**

Whilst both species were rarely captured in their nonpreferred habitat (5% *M. natalensis* inside houses; 23% *R. rattus* outside houses), evidence of RhB consumption when bait was in nonpreferred habitat was high for both species (50% *M. natalensis*; 57% *R. rattus*), indicating frequent movement to nonpreferred habitats. Whilst *R. rattus* movement distances were consistent with previous studies (maximum 81 m), within our village context, *M. natalensis* moved further (maximum 132 m) compared to previous published studies. Although bait consumption rates varied seasonally, we found no evidence that utilization of nonpreferred habitat varied seasonally. *M. natalensis* females moved into houses more frequently than males, whilst immature *R. rattus* moved outside houses more than mature individuals.

**CONCLUSION:**

These findings highlight the dynamic movement patterns of commensal rodents with implications for control and disease transmission. © 2024 The Author(s). *Pest Management Science* published by John Wiley & Sons Ltd on behalf of Society of Chemical Industry.

## Graphical abstract

Two rodent pest species, *Mastomys natalensis* and *Rattus rattus*, demonstrate persistent use of nonpreferred habitats. Although both were rarely caught in their nonpreferred habitat, a high proportion of animals consumed Rhodamine B bait when it was placed in their nonpreferred habitat.

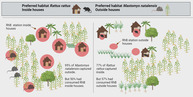

## 1 INTRODUCTION

Rodents, comprising over 40% of all mammalian species, are among the most adaptable and prolific animals on the planet.^1^, ^2^ Their adaptability allows them to thrive in a wide range of environments, from forests and grasslands to urban centres.^3^, ^4^, ^5^ Although rodents play vital ecological roles, such as seed dispersal and soil aeration, rodents also cause serious impacts on agriculture, stored food and health.^6^, ^7^, ^8^, ^9^, ^10^, ^11^ Rodent pest species include synanthropic species, which live in close association with human dwellings, inhabit agricultural and peridomestic areas, and some species can use multiple habitat types.^4^, ^11^, ^12^ Rodents are known to move between different habitats and this may be critical for understanding their spatial population dynamics. Additionally, their movement across diverse habitats can significantly influence the spread of diseases. Rodents can carry infectious agents between fields, homes and other settings, potentially creating a network of disease transmission that poses risks to human health.^13^ Rodents are known carriers of numerous zoonotic diseases such as Lassa fever, plague and leptospirosis, which have serious public health implications. Understanding rodent movement patterns is therefore crucial for identifying potential links between different environments that could facilitate disease transmission, and for designing effective rodent control strategies and implementing measures for disease prevention.

*Mastomys natalensis* and *Rattus rattus* are serious rodent pests in Sub‐Saharan Africa, with different ecology and habitat preferences. Several studies have extensively explored the ecology and movement patterns of *M. natalensis* in crop fields, which are their preferred habitats.^14^, ^15^ These studies indicate that *M. natalensis* exhibit seasonal fluctuations in population density, often driven by food availability. During the crop‐growing season, *M. natalensis* can multiply rapidly.^14^, ^16^, ^17^ Absolute population abundance studies using capture–mark–recapture (CMR), have typically concentrated on movements of rodents solely in agricultural fields, often depicting movement and home range of rodents within the fields.^18^ However, there has been limited exploration of rodent movement between houses, peridomestic areas and crop fields surrounding houses for species such as *M. natalensis* and *R. rattus*. Such studies, if available could be useful in revealing the potential risks of parasites and diseases transmission across the houses and surroundings in rural areas.^19^ Also, few studies have examined the ecology and movement of *M. natalensis* in areas immediately adjacent to houses, which are nonpreferred habitats.^20^, ^21^

The literature has shown that *R. rattus* tend to prefer residential areas,^22^ but there is limited information on their movement from inside houses to areas surrounding houses and other habitats. Understanding their movement is essential, because despite their preference for indoor habitats *R. rattus* may occasionally venture outside to find additional food sources, regulate their body temperature, or escape from competition within the house.^10^, ^23^ This movement can significantly influence the spread of diseases and the effectiveness of control strategies.

This study aimed at exploring the frequency of movement of *M. natalensis* from crop fields close to villages and peridomestic areas to inside houses, and *R. rattus* from inside houses to the peridomestic areas and crop fields close to villages. We hypothesized that the frequency of movements would vary between seasons and depend on the age and sex of individuals. We used Rhodamine B (RhB) baits, with baits placed outside in some villages and inside in others. RhB is a nontoxic dye that can be observed on the nose and mouthparts using an ultraviolet torch immediately after consumption. After 24 h, it also can be detected in the whiskers under a fluorescent light microscope and remains detectable for 3 months.^24^, ^25^

## 2 MATERIALS AND METHODS

### 2.1 Study area

**Figure 1:**
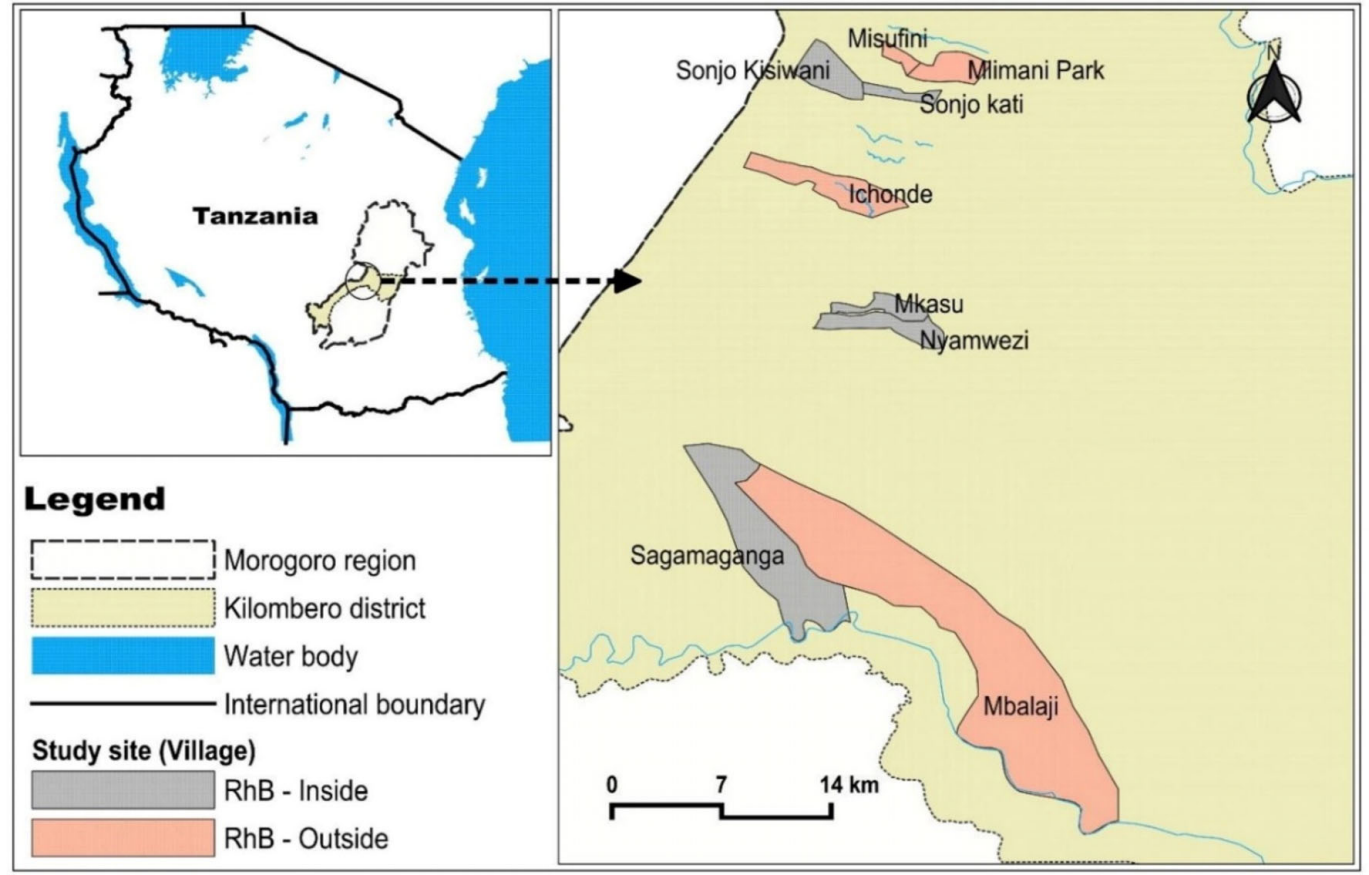
Map of Tanzania showing study areas in Kilombero District, Morogoro Region.

The study was conducted in Kilombero District, Morogoro Region, Tanzania. The district covers 14 918 km^2^ and is located on the western side of Morogoro Region, lying between latitudes 7°40 and 9°21S and between longitudes 35°20 and 37°48E, with an average elevation of 208 m above sea level (Fig. 1). A large part of Kilombero district is situated in a floodplain area, which is indispensable for agriculture and livestock keeping. The main crops cultivated in Kilombero district are rice and sugarcane.^26^ The climate of the study area is characterized by wet and dry seasons, which are further distinguished into four sub‐seasons: hot wet (December to March), cool wet (April–June), cool dry (July–August) and hot dry (September–November). The area receives between 1200 and 1800 mm of rainfall per year and temperatures range from 26 to 32 °C.^26^

This study encompassed nine representative villages (Fig. 1): Misufini, Ichonde, Mlimani Park, Sonjo Kisiwani, Mbalaji, Nyamwezi, Mkasu, Sagamaganga and Sonjo Kati. The surrounding areas of each village exhibited a mix of habitats including crop fields, fallow lands or vegetated areas that provided potential good habitats for rodents. The environments of these villages allowed us to investigate both the movement of *R. rattus* from inside households to external areas and the movement patterns of *M. natalensis* from external areas to inside houses.

### 2.2 Rhodamine B baiting and rodent trapping

The RhB bait was used to explore movement patterns of *M. natalensis* and *R. rattus* within and around the villages. Baiting and subsequent trapping was repeated on four occasions aligned with the agricultural seasonal cycle (crop stages) of the farms as follows: vegetative stage (June 2022), crop harvesting (August 2022), post‐harvesting (November 2022) and sowing period (February 2023). The agricultural seasonal cycle follows rainfall patterns. The bait used contained a conventional mixture of 1 kg maize flour and peanut butter, 2 g RhB (Sigma‐Aldrich, Burlington, MA, USA) and a few drops of water to make the mixture intact.^8^, ^15^ We used bamboo cylinders (≈4–5 cm radius and 20 cm length) for placement of the RhB bait to prevent consumption by nontarget species such as chickens. Bait stations were filled with ≈20 g RhB bait and checked for signs of consumption the next morning. Baiting was carried out for 2–3 days and replenished to the same amount (20 g) every morning. Bait was frequently consumed on the first night. In June 2022 and August 2022 pre‐baiting (bait with no RhB) was carried out for two consecutive days before the distribution of RhB baits. Again, at many bait stations the bait was consumed on the first night. Thus, we had little evidence of neophobic behaviour and from November 2022 onwards, no pre‐baiting was conducted.

In order to understand the movement of the two rodent species, Misufini, Mlimani Park, Mbalaji and Ichonde villages had RhB bait placed outside houses, close to water taps and Mkasu and Nyamwezi villages had RhB bait placed inside houses. Villages close together were allocated to the same treatment to avoid uncertainty over whether individuals had eaten RhB inside or outside. Given the apparent habitat preferences of the two species, the study was designed to explore to what extent the two species visited nonpreferred habitats. Baiting and trapping was repeated on four occasions aligned with the agricultural seasonal cycle (crop stages) of the farms as follows: vegetative stage (June 2022), crop harvesting (August 2022), post‐harvesting (November 2022) and sowing period (February 2023). The agricultural seasonal cycle follows rainfall patterns. In February 2023, to increase the number of villages with RhB bait inside houses, we extended the study by including three additional villages: Sagamaganga, Sonjo Kati and Sonjo Kisiwani.

Villages with RhB bait placed outside houses close to water taps had between two and three bait stations. As water‐taps were consistently located in peridomestic areas of each village, we chose to use water‐taps to standardize the location context of outside bait stations. Moreover, water‐taps may act as an important source of water for both species and may be interesting as potential hotspots for transmission of certain diseases such as leptospirosis.^27^, ^28^ Within each village up to five water‐taps were chosen to be distributed across the village. Villages with RhB bait placed inside houses had three RhB bait stations (kitchen, food store and bedroom) placed inside each of 10 chosen households distributed throughout the village.

Rodent trapping commenced 1 day after the removal of RhB bait. Trapping was conducted for four consecutive nights, except for two field trips at vegetative stage (June 2022), and at crop harvesting (August 2022) when trapping was carried out for 3–6 nights. Trapping points outside were located close to suitable vegetation and the minimum distance between trapping points was 10 m. For villages with RhB bait placed outside, trapping was conducted as follows: four trapping points close to the RhB bait station (<30 m in 97% of cases), four houses (two houses close to the water‐tap station and two further away), with a trapping point inside the house and one in the peridomestic area of the house, and six trapping points in crop fields/vegetation areas. As part of another study, additional trapping points were located in houses located across the village, and animals captured in these locations were also included.

**Figure 2:**
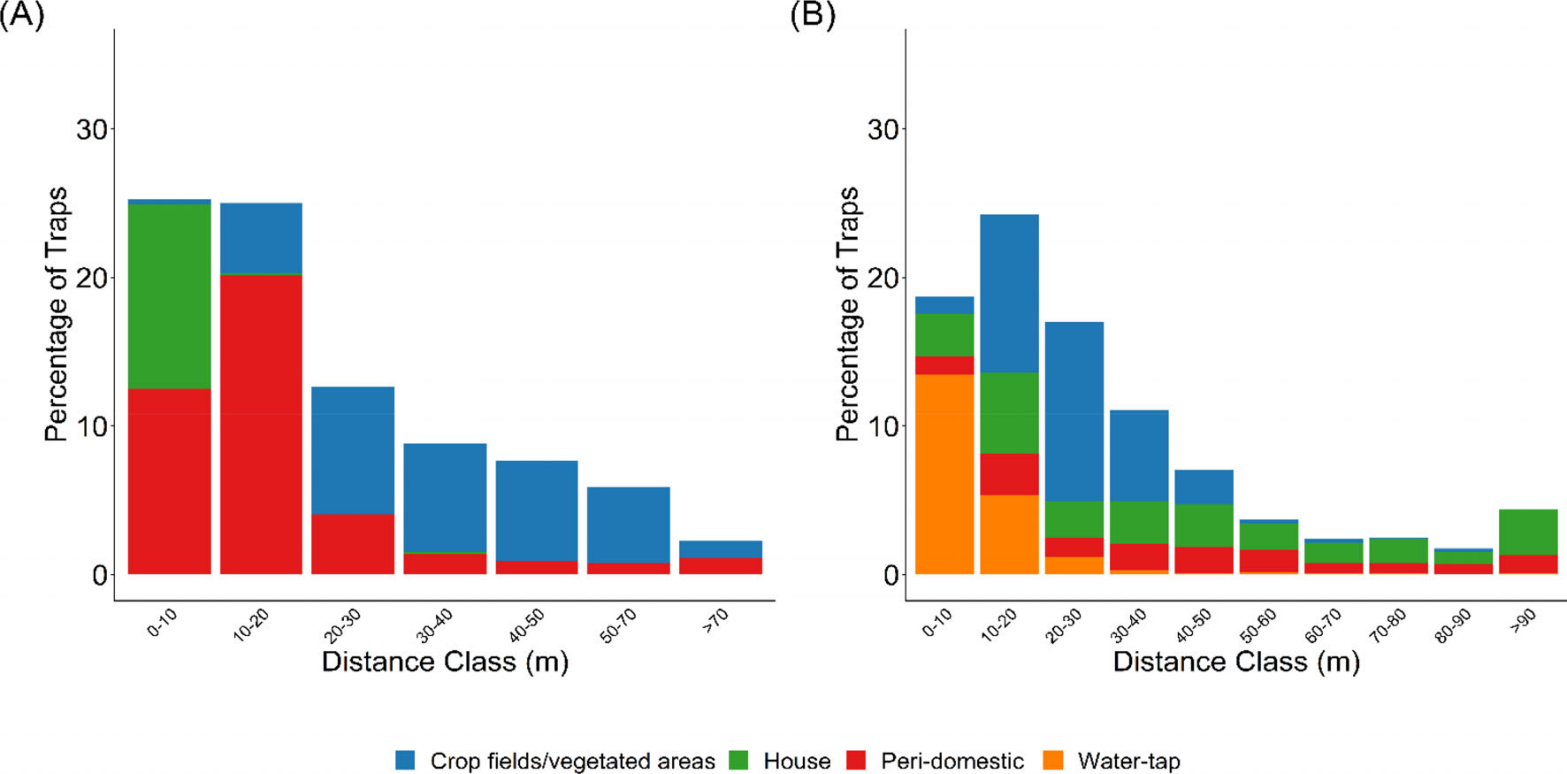
Percentage of traps located at different distances from the closest Rhodamine B (RhB) station, split by habitat type for the trap location, for villages where RhB bait stations were positioned (A) inside houses, and (B) outside, near water‐tap stations.

For villages with RhB bait placed inside houses, traps were set in the following habitats: one trapping point set inside each house with RhB bait, four trapping points in peri‐domestic areas of the house and four trapping points in crop fields/vegetated areas around the houses. The trapping scheme resulted in trapping locations with a range of distances from the closest RhB station (Fig. 2). As trap types vary in their ability to capture the two focus species, we deployed one Sherman (Tallahassee, FL, USA) and one modified box trap at each trapping point. However, owing to a shortage of modified box traps (locally made single capture live trap) at the beginning of the study, modified box traps were only set inside houses in June 2022 and August 2022. Subsequently, all locations had one of each trap type.

### 2.3 Collection of data on captured animals

The traps were inspected every morning before 10:00 h. Captured animals were removed from the traps and euthanized using halothane. Key morphometric and anatomical parameters such as body weight, head‐to‐body length, tail length, hind foot length and ear length were recorded, and species identification followed keys published previously.^29^ Sexual maturity status (immature or mature) was determined based on the reproductive status of the animal.^30^ For males we used scrotal testes, whereas for females we used perforated vagina, pregnancy by palpation and/or lactation. Each animal was examined for evidence of RhB consumption using an ultraviolet torch.^31^ Observation was done in the field, using hands to provide shade and enhance visibility of fluorescence. RhB fluorescence was frequently observed in the nasal, mouth and eye areas, and we assume that any fluorescence in these body parts of a captured animal was evidence of consumption of the bait within the same field session in the last 2–8 days. All analyses described below were based on recent consumption of RhB as assessed in the field.

### 2.4 Statistical analysis

In order to investigate what factors influenced the probability that an animal had recently consumed RhB bait, we used generalized linear mixed models (GLMMs), with binomial errors. As the local context may influence the frequency of movements, village was incorporated as a random effect to account for nonindependence. We employed an AIC‐based approach for covariate selection, with the best model having the lowest.^32^ We used the package glmmtmb to run GLMMs in R statistical software (R Core Team 2023).^33^, ^34^

### 2.5 Hypotheses and associated explanatory covariates

The probability that an individual animal will have consumed RhB depends on both opportunity and willingness to eat the bait. Distances moved by individuals and their willingness to enter nonpreferred habitat would influence how many animals have the opportunity to consume the bait. We expected rodents with home ranges close to the RhB stations to have greater opportunity and be more likely to consume the bait.^8^ We also expected consumption rates to be higher when RhB bait is in the preferred habitat for the species. Based on habitat preferences identified in previous trapping studies in similar contexts in Tanzania, we expected *R. rattus* to consume RhB bait more in the villages with RhB inside, and *M. natalensis* to consume RhB bait more in the villages with RhB outside.^8^, ^14^, ^22^ For each species, we used univariate GLMM to compare RhB consumption rates between villages with RhB bait placed outside or inside houses. We then used univariate GLMMs conducted separately according to the species and placement of the RhB stations (inside houses or outside) to explore whether consumption rates differed between individuals caught near or far from an RhB station. Based on the minimum distance from place of capture to the closest RhB station, two distance variables were considered: (i) distance as a continuous covariate and (ii) a categorical distance covariate. For the categorical covariate, we classified each animal as caught ‘near’ an RhB station or ‘far’ from an RhB station. The threshold distances for this categorical distance covariate were based on reported home ranges from previous studies for each species, and were fixed to 25 m for *M. natalensis* and 50 m for *R. rattus*.^18^, ^26^, ^35^ Although we did not know what bait station an individual that had consumed bait had visited, we obtained minimum movement distances for such individuals by using the distance to the closest bait station.

Following these initial analyses, we used multivariate GLMMs to explore factors that may influence the frequency of movements between preferred and nonpreferred habitats. We considered covariates that may influence the probability of RhB consumption by rodents, by affecting either their willingness to enter a nonpreferred habitat and/or their propensity to consume the bait. Analyses were conducted separately for each species. The global model for each analysis included RhB bait placement (inside or outside), time of sampling (crop stage and seasons), capture location (preferred or nonpreferred habitat), sex and maturity status (Table 1). We also included several interactions to investigate specific hypotheses.

**Table 1:** Covariates included in the analysis of Rhodamine B (RhB) consumption probability and their definition

Variable	Definition
Distance (continuous)	Minimum distance (m) from place of capture to RhB bait station
Distance (categorical)	Categorical covariate for minimum distance (m) from place of capture to RhB bait station: *Mastomys natalensis*: ‘Near’ ≤ 25 m, ‘Far’ > 25 m; *Rattus rattus*: ‘Near’ ≤ 50 m, ‘Far’ > 50 m.
RhB bait placement	Location of RhB bait stations: inside or outside in the village.
Capture location	Preferred or nonpreferred habitat: *M. natalensis* preferred: crop fields/peridomestic; *R. rattus* preferred: inside houses.
Time of sampling	Season: dry or wet season. Crop stage: June (vegetative), August (harvesting), November (post‐harvesting), February (sowing).
Sex	Females or males.
Maturity status	Mature: Individuals with apparent explicit mature sexual conditions (scrotal for males; perforated vagina, lactating or pregnant for females)
	Immature: Other individuals

We hypothesized that consumption rates would be highest when RhB was in the preferred habitat and individuals were captured in the preferred habitat, and lowest when RhB was in the nonpreferred habitat but where individuals were captured in the preferred habitat. We therefore included a two‐way interaction between RhB bait placement and capture location. Resource availability may vary temporally, for example owing to crop stages, or spatially (e.g. between different habitats). This may affect willingness to enter nonpreferred habitats and/or propensity to consume the bait. For example, when RhB bait is in the preferred habitat consumption rate may vary temporally owing to changing resource availability and therefore changes in willingness to consume the bait. Likewise, consumption rates when RhB is in the nonpreferred habitat could vary temporally if willingness to enter nonpreferred habitat changes through the year, with the expectation that animals may be more likely to enter the nonpreferred habitat in periods of low resource availability in their preferred habitat. We therefore included an interaction between RhB bait placement and time of sampling.

Lastly, rodents also may move to find mating opportunities or to avoid intraspecific competition, and therefore opportunity to eat RhB may vary temporally and depend on sex and maturity status. We therefore considered a three‐way interaction between RhB bait placement, sex and maturity.

Backward selection was conducted using the drop1 function based on AIC. Covariates were removed from the global model one by one, starting from the most complex interaction, until removal of further covariates resulted in an increase in the AIC.

## 3 RESULTS

### 3.1 Habitat and rodent community composition

**Figure 3:**
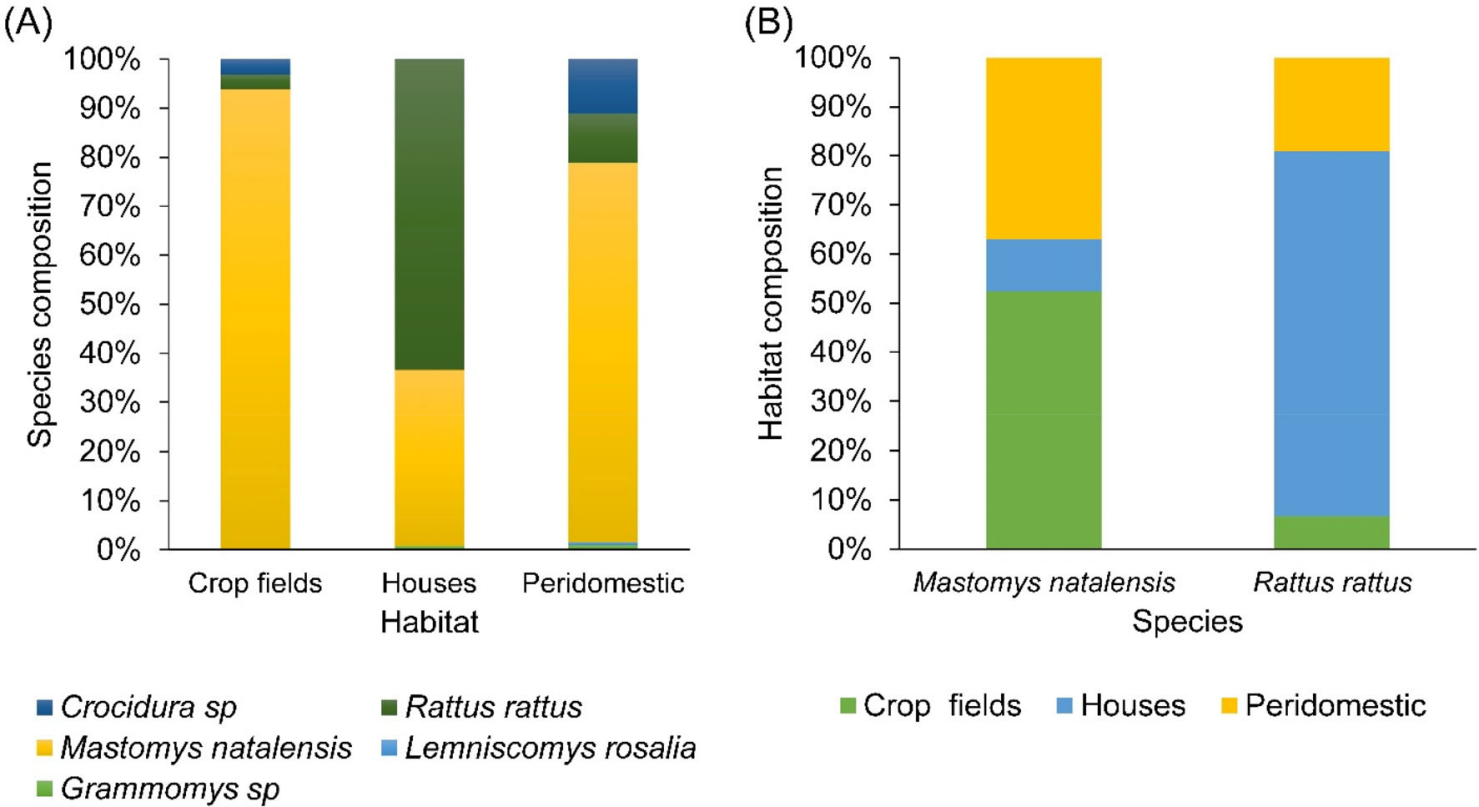
Composition of small mammal community in different habitats (A), and percentage of captures in different habitats for *Mastomys natalensis* and *Rattus rattus* (B).

In total, 552 individual small mammals were captured comprising of five species: *Mastomys natalensis* (75%), *Rattus rattus* (19%), *Grammomys dolichurus* (0.54%), *Lemniscomys rosalia* (0.18%) and *Crocidura* sp. (5.2%), with the species composition differing significantly between habitats [Fig. 3(A)]. As expected, *M. natalensis* and *R. rattus* were predominantly captured in crop fields and houses, respectively; with 52.4% of the *M. natalensis* captured in crop fields and 74.2% of *R. rattus* caught inside houses [Fig. 3(B)].

### 3.2 RhB consumption rates, use of nonpreferred habitats and movement distances

We found strong evidence of frequent movement to and from the nonpreferred habitat for both *R. rattus* and *M. natalensis*. Consumption rates of RhB (percentage of animals that had recently consumed bait) were high for *M. natalensis* (67% in villages where RhB bait was placed outside; 50% in villages with RhB inside) and *R. rattus* (57% in villages where RhB bait was placed outside; 57% in villages with RhB inside). For both species there was no evidence that consumption rates were higher when bait was located in the preferred habitat, with the intercept only model having the lowest AIC, indicating no difference in consumption rates between villages with RhB inside and villages with RhB outside (ΔAIC = +1.6 for *M. natalensis* and *R. rattus*).

**Figure 4:**
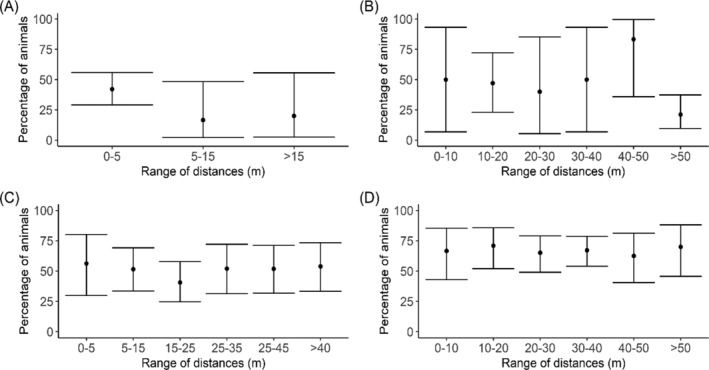
Percentage of animals with evidence of Rhodamine B (RhB) consumption according to minimum distance between capture location and RhB bait station for (A) *Rattus rattus*‐RhB bait placed inside houses, (B) *R. rattus*‐RhB bait placed outside houses close to water taps, (C) *Mastomys natalensis*‐RhB bait placed inside houses and (D) *M. natalensis*‐RhB bait placed outside houses close to water taps. Owing to the low numbers of *R. rattus* caught outside, minimum distances were grouped into fewer distance bands for villages where RhB was placed inside. Bars represent 95% confidence intervals.

There also was relatively little evidence of declining consumption rates with increasing distance between capture location and the closest RhB station (Fig. 4). The observed maximum distance moved was 132 m for *M. natalensis* and 81 m for *R. rattus*, noting that these distances are based on the shortest distance from the point of capture to a RhB station and are therefore conservative. The GLMM analyses only revealed a negative effect of distance on consumption rates for *R. rattus* when RhB was placed inside houses, with the model with distance as continuous covariates having the lowest AIC. For all other analyses, the intercept only model had the lowest AIC indicating no effect of distance (Table 2).

**Table 2:** Results for generalized linear mixed model analyses to investigate the effect of distance (m) from Rhodamine B (RhB) station on RhB consumption

	*Rattus rattus*_RhB inside^†^	*R. rattus*_RhB outside^‡^	*Mastomys natalensis*_RhB inside^§^	*M. natalensis*_RhB outside^¶^
Intercept	108.8	87.0	231.4	253.6
Distance continuous	107.4	88.8	233.28	255.2
Distance category	108.0	87.9	233.3	253.7

Aikake information criterion (AIC) for intercept only model as well as distance continuous and distance categorical. Information corresponds to results from AIC model selection when using distance continuous, distance categorical and intercept only model.

^†^ *R. rattus*_RhB placed inside houses.

^‡^ *R. rattus*_RhB placed outside houses close to water taps.

^§^ *M. natalensis*_RhB placed inside houses.

^¶^ *M. natalensis*_RhB place outside close to water taps.

### 3.3 Factors influencing RhB consumption and use of nonpreferred habitats

**Figure 5:**
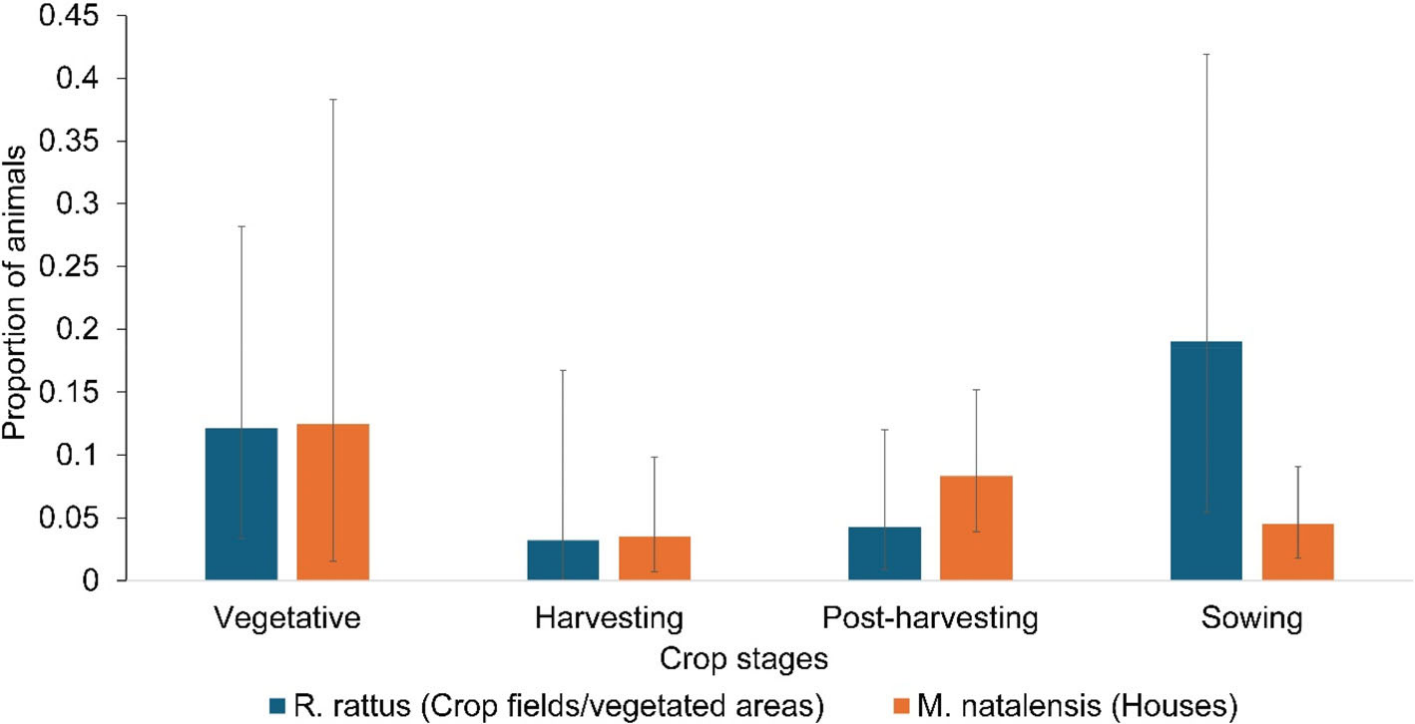
Proportion of individuals caught in nonpreferred habitat according to crop stage (crop fields/vegetated areas for *Rattus rattus*; houses for *Mastomys natalensis*). Bars represent 95% confidence intervals.

Both rodent species were caught in their nonpreferred habitat in each field season, irrespective of crop stage (Fig. 5). The proportion of animals caught in the nonpreferred habitat did not vary between crop stage (*M. natalensis*: *P* = 0.22; *R. rattus*: *P* = 0.09).

The final models from the multivariate GLMM analyses on RhB consumption are given in Table 3. Consistent with the results above suggesting frequent movement to nonpreferred habitats, we found little evidence that individuals were most likely to consume RhB bait when the bait was located and the individual was caught in the preferred habitat for the species, with no interaction between capture location and RhB bait placement in the final models. However, *R. rattus* were overall more likely to consume the bait when it was placed inside (Table 3).

**Table 3:** Coefficients estimates and 95% confidence intervals from the final competitive model(s) for *Rattus rattus* and *Mastomys natalensis*

Variables	*R. rattus*	*M. natalensis*
Intercept	−2.52 (−5.00, −0.03)	2.08 (1.08, 3.08)
Position of RhB bait (outside)	0.05 (−2.96, 3.06)	−1.04 (−2.38, 0.30)
Capture location (preferred)	1.04 (−0.08, 2.16)	
Crop stage (vegetative, harvesting, sowing)	2.30 (1.07, 3.53)	−1.03 (−2.21, 0.15)
	2.72 (1.43,4.00)	−0.12 (−0.86, 0.63)
	−0.75 (−3.05, 1.55)	−1.71 (−2.34, −1.09)
Sex (males)		−1.34 (−2.04, −0.63)
Maturity status (mature)	1.04 (−0.21, 2.30)	
Position of RhB bait: Sex (males)		0.84 (−0.13, 1.82)
Position of RhB bait: Maturity status	−1.58 (−3.40, 0.24)	
Sex: Maturity status		

**Figure 6:**
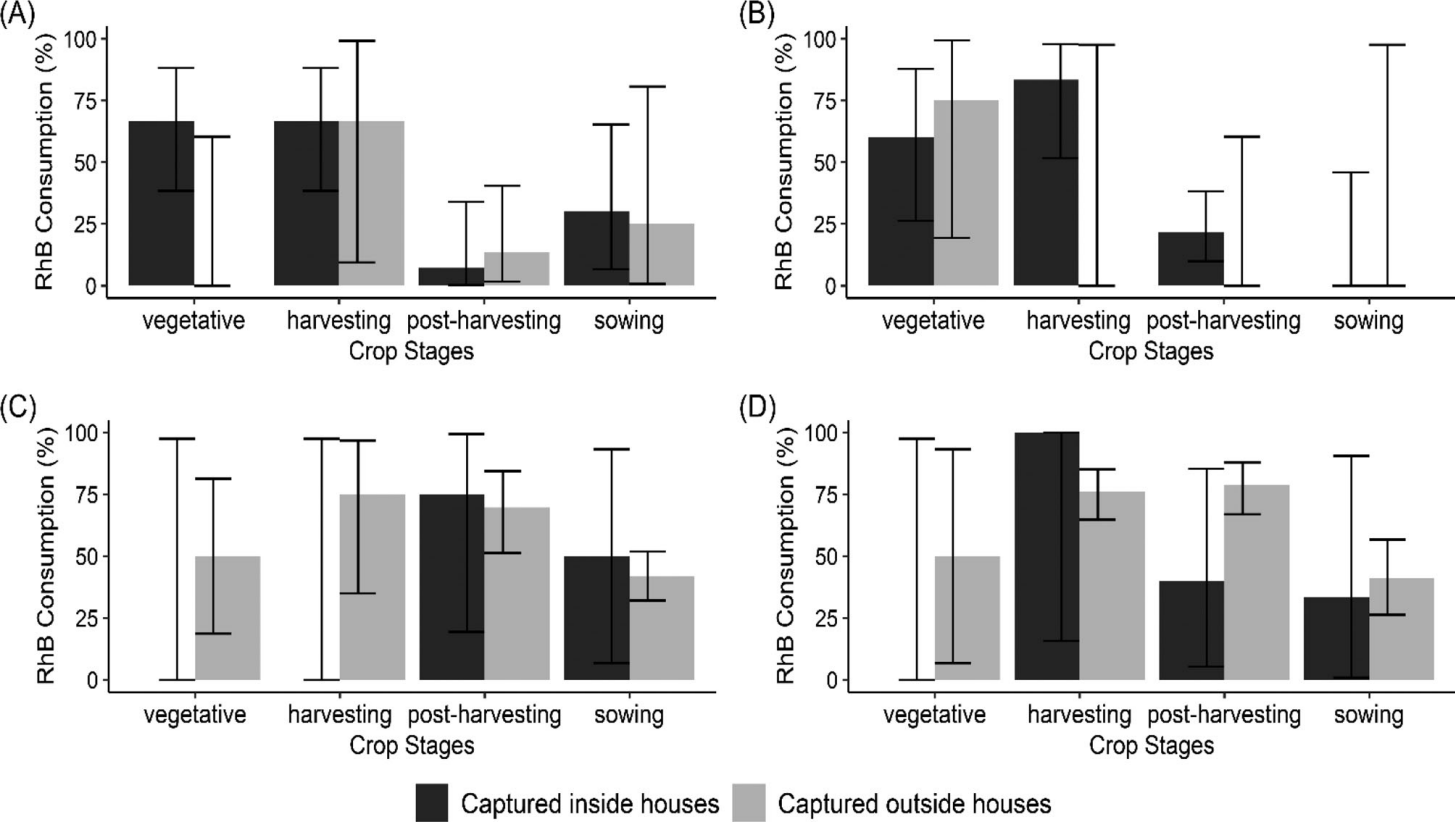
Percentage of animals with evidence of Rhodamine B (RhB) consumption by crop stage for each species according to capture location for (A) *Rattus rattus*_RhB placed inside houses, (B) *R. rattus*_RhB placed outside houses close to water taps, (C) *Mastomys natalensis*_RhB placed inside houses, and (D) *M. natalensis*_RhB place outside close to water taps. Dry season: harvesting and post‐harvesting. Rainy season: vegetative and sowing. Bars represent 95% confidence intervals.

We observed strong evidence of temporal changes in RhB consumption rates (Fig. 6) indicated by a larger change in AIC (*R. rattus* ΔAIC = + 25.4; *M. natalensis* ΔAIC = +31.58). However, there was no evidence of an interaction between RhB bait placement and time of sampling (Table 3), indicating that these temporal changes were similar whether RhB bait was located in the preferred or nonpreferred habitat [comparing seasonal patterns for black bars (*R. rattus*) in Fig. 6(A and B); and grey bars (*M. natalensis*) in Fig. 6(C, D)]. For *M. natalensis*, consumption rates are particularly high in the harvesting and post‐harvesting season, whilst for *R. rattus* consumption rates were highest in the vegetative and harvesting seasons (Fig. 6; Table 3).

**Figure 7:**
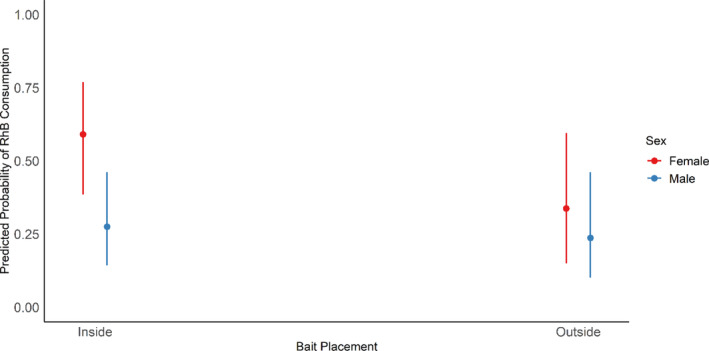
Predicted probability of having evidence of recent Rhodamine B (RhB) consumption for *Mastomys natalensis*, based on bait location and sex. Error bars represent 95% confidence intervals.

**Figure 8:**
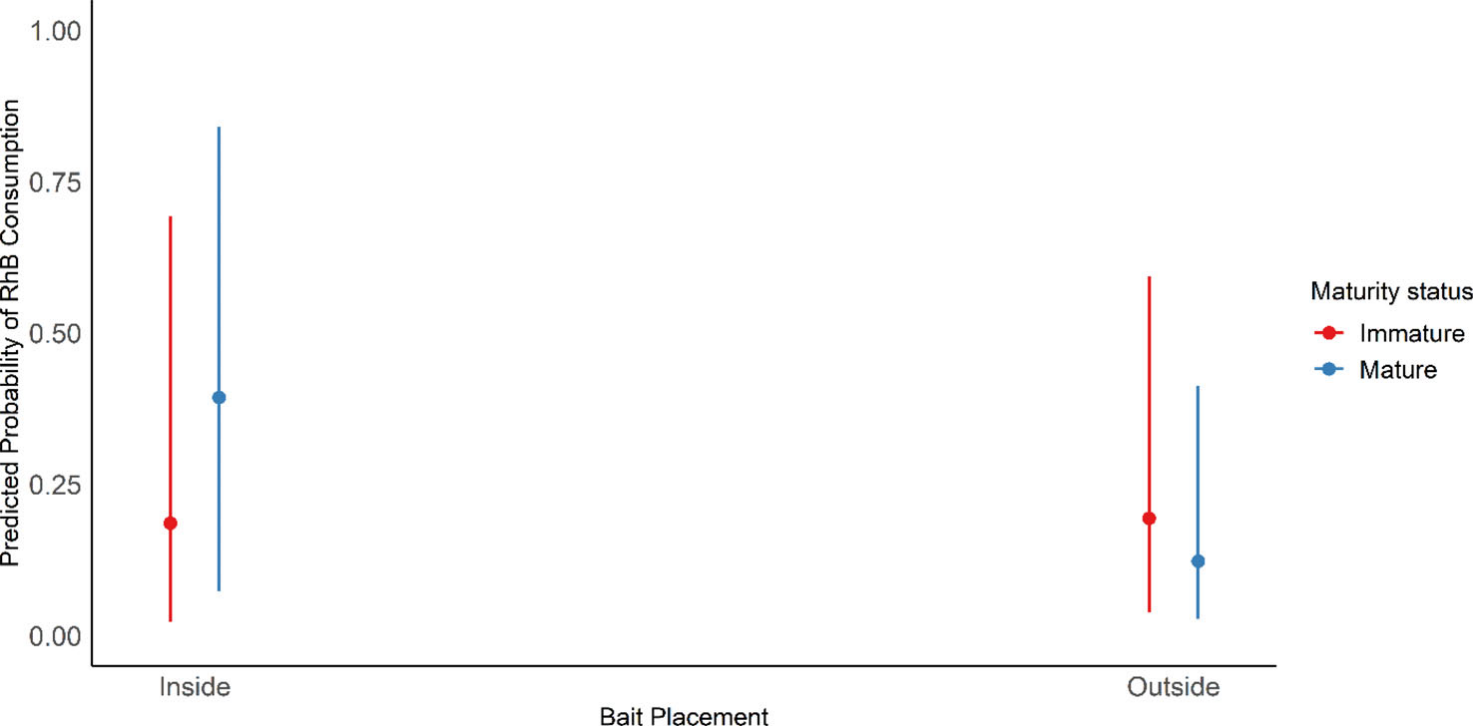
Predicted probability of having evidence of recent Rhodamine B (RhB) consumption for *Rattus rattus*, based on bait location and maturity status. Error bars represent 95% confidence intervals.

For both species, we found evidence that RhB consumption rates, and possibly the willingness to enter nonpreferred habitat, may vary between individuals, with an interaction between RhB bait placement and sex for *M. natalensis* and an interaction between RhB bait placement and maturity for *R. rattus* (Table 3). For *M. natalensis*, when RhB bait was in preferred habitat (outside), almost similar consumption rates were observed between males and females (Fig. 7). However, females had very high consumption rates when RhB bait was in the nonpreferred habitats (inside houses; Fig. 7), indicating frequent movement to the nonpreferred habitat, as well as a high willingness to consume the bait when they are there. For *R. rattus*, mature animals were more likely to consume RhB bait than immature animals when RhB was in the preferred habitat (inside houses; Fig. 8), suggesting that immatures are less willing to consume the RhB bait or that they may be excluded from bait within houses. However, mature animals are less likely to consume RhB located outside than when it is inside, whilst immature animals have similar consumption rates (Fig. 8). This suggests either that immature individuals move more frequently to nonpreferred habitat, or that mature animals are less likely to consume the baits when outside than inside.

## 4 DISCUSSION

We used RhB bait to explore movements of *M. natalensis* and *R. rattus* in village habitats, focusing on evidence of movements between their preferred and nonpreferred habitat across different crop stages. *M. natalensis* were mostly captured outside and *R. rattus* were mostly captured inside houses; this is consistent with habitat preferences reported in previous studies.^8^, ^15^, ^16^, ^18^ However, we found new evidence that both species made frequent movements to their ‘nonpreferred’ habitat. Thus, even though only 23% of *R. rattus* were caught outside houses, 57% of *R. rattus* in villages where RhB bait was located outside had evidence of consumption, whilst for *M. natalensis* only 5% of animals were caught inside houses but 50% of animals captured in villages with RhB bait inside had consumed the bait. We found little evidence supporting our hypothesis that the frequency of movements would vary temporally and depend on crop stage. However, we did find evidence suggesting that certain categories of animals may make more frequent movements.

Compared to other studies, our finding of higher rates of movement between preferred and nonpreferred habitats and distances that were longer than the expected home ranges, especially for *M. natalensis*, could be to the result of different contexts and/or different methodologies. CMR studies conducted in cropping and fallow areas have shown relatively small home ranges for *M. natalensis*, with regular movements of ≈25 m.^16^, ^18^ Notably, in our study *M. natalensis* regularly made movements which were longer than expected based on these previously recorded home ranges. Also, we did not find evidence of a decline in RhB consumption rates with distance, suggesting that in this context of mixed habitats close to village their movements span greater distances. This would infer that *M. natalensis* have a broader exploration and utilize a wider range of habitats. For *R. rattus*, although they frequently moved from houses into surrounding areas, there was some evidence of decline in RhB consumption rates at locations further from bait stations (>50 m), indicating that most movements are of shorter distance. This supports previous studies that have documented relatively limited movement ranges for *R. rattus* in various contexts. For example, studies conducted in crop fields have shown that *R. rattus* tend to occupy relatively small home ranges, with movements often constrained within agricultural landscapes.^36^, ^37^ Whilst in urban environments, *R. rattus* also typically exhibit restricted movements, with studies reporting movement ranges of around 20–50 m.^38^ As *R. rattus* are primarily commensal with humans, movements may be restricted within human settlements owing to food storage facilities, and residential buildings providing ample food.^22^ Given that in this study we found larger than expected movement ranges, future studies should deploy traps at greater distances from RhB bait stations to understand the extent of movement fully. To understand movements and habitat use by these two species within village areas, it would also be useful to have RhB stations within different types of habitat as the placement of RhB bait stations near water taps could have influenced our results.

The results of our study have some similarities and some key differences from the study of *R. rattus* and *M. natalensis* in areas surrounding villages in several African countries, which used telemetry to explore movements^15^ The telemetry study found that *M. natalensis* can exhibit extensive movements between cropping areas and nearby habitats, whilst in some villages a proportion of *R. rattus* locations were located outside houses, supporting our results that both these rodent species can move to their nonpreferred habitat.^15^ The telemetry study documented temporal variation in movement patterns, particularly noting an increased frequency of *M. natalensis* entering houses during the post‐harvest period in Namibia.^15^ Likewise, a CMR study discovered that in rice fields, *M. natalensis* travelled shorter distances during the dry season's maturity stage, whereas in fallow fields, distances were shorter during the wet season's transplanting stage.^18^ *R. rattus* also have showed seasonal variations in movement patterns in urban environments.^37^ In our study, we observed similar seasonal patterns in consumption of bait irrespective of whether the bait is in the preferred or the nonpreferred habitat. We therefore argue that this strong seasonal variation is more likely to be related to changes in willingness to eat the bait rather than changes in willingness to enter nonpreferred habitats. Thus, we found *M. natalensis* entering houses and *R. rattus* moving to outside areas consistently across all crop stages (corresponding to dry and wet season). Telemetry studies can provide detailed movement patterns of a few individuals but the number of rodents that can be tracked is typically limited. Moreover, telemetry studies also can have problems with signal. Using RhB methodology can allow for a more comprehensive assessment of movement across a larger population of rodents and is cheaper than telemetry. However, our finding that willingness of rodents to eat the bait may vary between seasons means that care must be taken when making comparisons and interpreting results. Whilst we had no evidence that pre‐baiting increased consumption rates in our study system, pre‐baiting may help to reduce such seasonal variation.

*Mastomys natalensis* showed higher rates of RhB consumption during the post‐harvesting and harvesting periods, whilst *R. rattus* showed higher rates of RhB consumption during the vegetative and harvesting stage. Both species showed relatively low rates of RhB consumption in the sowing period. High bait consumption rates may be linked to seasons with potentially low resources in preferred habitats, which suggests a link between bait acceptance and seasonal resource availability.^16^, ^38^

For both species, variation in RhB consumption rates between different categories of individual may reflect differences in willingness to enter nonpreferred habitats. Female *M. natalensis* had very high consumption rate when RhB was inside (the nonpreferred habitats), suggesting that females may enter houses more often, possibly because males are more dominant in the preferred habitats and outcompete females for resources.^39^ For *R. rattus*, we found evidence that immature animals moved to nonpreferred habitat more than mature individuals. We suggest that immature *R. rattus* might be excluded from bait within houses owing to the presence of aggressive adults, and that this competition may increase their movements to outside areas compared to adults.

The implications of our findings extend beyond rodent ecology to pest management and disease transmission. The longer than expected movements by *M. natalensis* carries significant implications. These extended movements reflect a high level of adaptability, allowing the species to traverse a variety of habitats and access a broader range of resources. This adaptability could lead to wider geographical distribution, making *M. natalensis* a more persistent agricultural pest as it can colonize new areas with ease. Moreover, the frequency of movement by both *M. natalensis* and *R. rattus* to their nonpreferred habitats increases the potential for interactions between the two species, potentially leading to competition for resources where their habitats overlap. Furthermore, increased movement distances implies greater interaction with other rodent species and human encounters that could increase pathogens transfers and disease transmission. Owing to these observed movements by *M. natalensis* from outside to inside houses, it is therefore important to ensure that rodent management in houses also involves control of rodents within the area surrounding the homestead. By understanding the spatial dynamics of rodent populations within human‐dominated landscapes, we can develop more effective rodent management plans and proactive measures to protect public health.^6^, ^40^

## CONFLICT OF INTEREST STATEMENT

The authors declare that they have no conflicts of interest regarding this article.

## AUTHOR CONTRIBUTIONS

HM conducted field data collection, data analysis and wrote original draft manuscript. ME assisted in the data analysis and reviewed the original draft. SB assisted fieldwork and reviewed the original drafts. AP reviewed the original draft. RM, AAR and AM supervised the research and reviewed manuscripts. MS assisted in data collection. ST contributed in designing protocol for field data collection, analysis and reviewed the original drafts. All authors read and approved the final version of the manuscript for submission.

## ETHICAL STATEMENT

The study followed regulations related to working with communities and seeking consent from household owners, as stipulated in Research Regulations and Ethics by the Directorate of Research and Postgraduate Studies of Sokoine University of Agriculture, Tanzania. Animal trapping and handling followed the guidelines of the American Society of Mammalogists (ASM) for the use of wild mammals in research and education (Sikes and Animal Care and Use Committee of the American Society of Mammalogists, 2016).

## Data Availability

The data that support the findings of this study are openly available on Open Science Framework (OSF): https://doi.org/10.17605/OSF.IO/VPYZH.
